# Sense of Coherence and Physical Health. A Review of Previous Findings

**DOI:** 10.1100/tsw.2005.85

**Published:** 2005-08-25

**Authors:** Trine Flensborg-Madsen, Søren Ventegodt, Joav Merrick

**Affiliations:** ^1^ The Quality of Life Research Center, Teglgårdstræde 4-8, DK-1452 Copenhagen K, Denmark; ^2^ Nordic School of Holistic Medicine, Teglgårdstræde 4-8, DK-1452 Copenhagen K, Denmark; ^3^ Quality of life Research Clinic, Teglgårdstræde 4-8, DK-1452 Copenhagen K, Denmark; ^4^ National Institute of Child Health and Human Development, Faculty of Health Sciences, Ben Gurion University of the Negev, Beer-Sheva, Israel; ^5^ Center for Multidisciplinary Research in Aging, Faculty of Health Sciences, Ben Gurion University of the Negev, Beer-Sheva, Israel; ^6^ Office of the Medical Director, Division for Mental Retardation, Ministry of Social Affairs, Jerusalem, Israel

**Keywords:** Antonovsky, sense of coherence, physical health, public health, human development

## Abstract

The aim of this paper is to systematically review the available scientific publications published concerning the association between the sense of coherence (SOC), designed by Aaron Antonovsky (1923-1994), measured with the scales SOC-29 or SOC-13, and different aspects of health. The study is descriptive and integrates more than 50 scientific publications. The results are divided into the categories: Physical health; biological measures; psychological measures; health measures incorporating psychological aspects; stress; and behavioural aspects. The conclusion from this review is that SOC is highly associated with psychological aspects, including stress and behavioural aspects when SOC is operationalized with the prevailing scales. However, we were unable to show a strong association between SOC and physical health that Antonovsky had predicted. Therefore, we conclude that the SOC scale can only serve as a predictor for health that is measured by incorporating psychological aspects, while it is not capable of explaining physical health that is measured only by means of physical terms.

## INTRODUCTION

In 1979 Aaron Antonovsky (1923-1994), who was affiliated with the Faculty of Health Sciences at the Ben Gurion University of the Negev, published a book called “Health, Stress and Coping”[1], which presented a theoretical model designed to advance an understanding of the relationship between stressors, coping and health. This model was more thoroughly and systematically developed in the book, which appeared in 1987, called “Unraveling the Mystery of Health. How People Manage Stress and Stay Well”[2], whose appendix contained the sense of coherence scale. The theory of the sense of coherence (SOC) defined by Antonovsky argued that a person’s SOC consists of the three dimensions defined as comprehensibility, manageability and meaningfulness with the argument that SOC is associated with both physical and psychological health. This is due to the fact that a strong SOC should lead a person to engage in behaviours which promote health and which also affect the endocrine and immunological systems, therefore mobilizing appropriate bodily resources[3]. Antonovsky wrote: “My hypothesis, then, is that the strength of the SOC has direct physiological consequences and, though such pathways, affects health status”[2].

Antonovsky's Sense of Coherence (SOC) scale has been used in several studies to verify the hypothesis that a person's sense of coherence is strongly associated with his health.. Unless mentioned, the studies in this review have used one of the original scales (SOC-29, SOC-13), but at least 15 different versions exist with different scoring alternatives[4,5]. The scales are constructed so that a high score indicates a high degree of sense of coherence. Several studies have shown that SOC is correlated with different kinds of health measures. However, most of these studies investigate psychological health, or they incorporate psychological aspects in their health measures, but very few studies investigate the direct association between SOC and physical health.

This paper is concerned with the question of the association between sense of coherence and physical health. The major purpose of this study is to present the available evidence that has been published regarding the association between SOC and different varieties of health measures in order to reveal an overall picture of the findings.

## METHODS

In order to identify studies that recorded the exposure SOC and outcome (health measures) of interest, we searched in the database PubMed/Medline (http://www.ncbi.nlm.nih.gov/entrez/query.fcgi). This review includes more than 50 scientific publications. Most of the studies were cross-sectional, although some longitudinal studies were found, and the study designs were quantitative including some intervention studies. The results were divided according to the following sections: Physical health; biological measures; psychological measures; health measures incorporating psychological aspects; stress; and behavioural aspects. The correlations found according to the physical health and biological measures are shown in Table 1.

## RESULTS

### Physical Health

Among the studies that investigated the connection between SOC and physical health was a prospective Israeli study investigating the effects of SOC on the health of men and women in two kibbutzim in Israel. They found quite contradictory results. Where the SOC had no significant effect on women’s health, or the functional ability among men, the physical well-being among men, however, was significantly affected by SOC[6]. A Danish study showed that poor SOC was associated with poor self-rated health and a high prevalence of symptoms among all age groups, with high prevalence of long-term illness among respondents older than 50 years of age[7]. In addition, a German cross-sectional study of a large community sample found a high SOC associated with both a lower extent of subjective body complaints, somatoform symptoms and with minor health-related problems in daily living[8]. A prospective study involving 107 patients with chronic pain investigated the value of SOC as a predictor of the response

**TABLE 1.**
**Correlations of SOC (SOC-29 or SOC-13) with physical health and biological measures.**

| 1st author | Ref. No. | Sample | N | Variable | r |
|---|---|---|---|---|---|
| **Physical health** | | | | | |
| **Carmel S** | 6 | Kibbutz members | 230 | Physical well.being (male) | 0.26 |
| | | | | Functional ability (male) | NS |
| | | | | Physical well.being (female) | NS |
| | | | | Functional ability (female) | NS |
| **Due EP** | 7 | Danish randomly sample from CPR-register | 2,352 | Self-rated health | 0.18-0.51[1] |
| **Schumacher J** | 8 | German community sample | 2,005 | Subjective body complaints | -0.35 |
| | | | | Somatoform symptoms | -0.34 |
| | | | | Stomach complaints | -0.20 |
| | | | | Joint complaints | -0.29 |
| | | | | Physical mobility | -0.28 |
| **Coe RM** | 11 | US male patients, 55+ | 377 | Perceived health | -0.458 |
| | | | | Long-term survival | NS |
| **Milanesi LC** | 16 | Japanese-American and Anglo-American women 60+ | 60 | Self-rated health | 0.48 |
| | | | | Health complaints | -0.39 - 0.48 |
| **Steiner A** | 10 | Residents of Santa Monica, California 75+ | 414 | Chronic medical conditions | 0.31 (!)[2] |
| | | | | Perceived health | 0.34 |
| | | | | Functional ability | 0.17 |
| **Biological measures** | | | | | |
| **Milanesi LC** | 16 | Japanese-American and Anglo-American women 60+ | 60 | Immunoglobulin | 0.33 – 0.46 |
| | | | | Cortisol level | NS |
| **Post-White J** | 17 | US outpatients with solid tumours | 38 | Natural Killer Cells | Sig.[3] |
| | | | | $T_H/T_S$ ratios | Sig.[3] |

1: The SOC scale was not used continuously; the sample was divided into different SOC groups in non-linear intervals. Correlations were measured for different age groups.
2: The correlation between chronic medical conditions and SOC was positive rather than negative as had been expected.
3: Regression Model ($p<0.05$)

to a brief pain management program. They found that reported pain intensity after the course was significantly associated with SOC subscale meaningfulness[9]. A study of 414 people over at least 75 years found a rather strong correlation between chronic medical conditions and SOC, but surprisingly the correlation was positive rather than negative as had been expected. On the other hand, they found that perceived health status and functional ability were both correlated positively with SOC[10]. Finally, a five year study that investigated the survival of a sample of 377 elderly men found a strong SOC was not associated with long-tem survival, but that declines in morale and perceived health status (not defined in details, which therefore might include psychological measures) were statistically significant related to declines in SOC scores over the five-year period[11].

Using a 16-item SOC questionnaire, a prospective study of 1,976 individuals found the initial level of SOC significantly associated with a subjective state of health four years later in both genders when adjustments were made for age[12]. However, another study found this causal effect of SOC on health only in females, while a cross-sectional association between SOC and health complaints was found also in males[13].

Using a three-item SOC questionnaire a prospective study of more than 20.000 participants found that a strong SOC was associated with a significant reduction in mortality from all causes, cardiovascular disease and cancer independent of age, sex and prevalent symptoms (except that no association was observed for cancer mortality in women[14]. And using the same three-item questionnaire another study found that SOC was associated with type 2 diabetes (RR=3.7) and insulin resistance (RR=4.2)[15].

## Biological Measures

Two rather small studies have shown interesting results according to immune function and overall health. A study of 60 Japanese-American and Anglo-American women aged over 60 years found that SOC was strongly correlated with self-rated health and two measures of health complaints together with immunoglobulin measures[16]. Another study including 22 patients and 16 control group patients found SOC as a predictor of increased natural killer cells and $T_H/T_S$ ratios[17].

## Psychological Measures

Since the majority of published studies investigated the connection between SOC and health incorporated psychological aspects in their health measures, it can be hypothesized that the SOC scale was contaminated with psychological facets. A prospective study (N=510) tested the possibility that SOC was confounded with emotionality. At first, they found that individuals with a strong SOC were significantly less physically symptomatic (with the health measure only including physical symptoms) at different levels of stress. However, the effect of SOC on health was removed once emotionality was taken out. The conclusion was that emotionality together with sex, prior symptoms and stress were found to predict physical symptoms one month after initial test administration and therefore presumed that it was not the SOC that predicted the symptoms. The study concluded that SOC failed to predict physical symptoms prospectively[18]. Another cross-sectional study found high negative correlations (average about –0,7) between SOC and negative affectivity (NA), with NA describing a personality trait with a consistent and pervasive way of experiencing, interpreting and reflecting negatively on the self and the world. The study suggested that SOC only measure the absence of neuroticism[19].

A study of nurses found high correlations between SOC and somatic anxiety (r= -0,619), psychic anxiety (r= -0,651) and hostility (r= -0,763) [20]. A study of technical designers showed a strong SOC associated with lower levels of psychosomatic symptoms and emotional exhaustion, where SOC seemed to have a moderating role on the relationship between perceived work characteristics and well-being[21]. A low SOC was found to be associated with increased prevalence of depression among people with rheumatoid arthritis[22]. A study investigating SOC and mental disorders found that SOC was significantly lower for people with mental disorders (neurotic patients and patients with depressive symptoms), and they also found significant correlations between SOC and self-rated health with SOC and symptoms intensity disappearing in the group of depressed people, in contradiction to the other two groups. They suggested that the protective function of SOC disappears in persons situated far away from the “health” pole of the health-disease continuum[23].

In a study of traffic accident victims, the SOC correlated negatively with the development of post-traumatic psychopathology, psychological disorders and anxious cognitions[24]. It was shown that hyperactive children have lower levels of SOC than a control group[25]. SOC has been shown to be associated with trait anxiety and current depression[26]. The personality trait of neuroticism has been shown to be correlated negatively, while extraversion and frustration tolerance correlated positively with SOC[24]. Reported longstanding psychiatric illness and utilization of psychotropic drugs were also found to correlate negatively with SOC (using a three-item SOC questionnaire)[27], while a study among minority women at risk for HIV infection showed a significant relationship between level of coherence and concerns, appraisals of threat and emotional distress[28].

## Health Measures Incorporating Psychological Aspects

In a cross sectional study investigating 148 people with a high-risk childhood, it was found that there was a strong correlation between SOC and the health measure, SCL-90, incorporating psychosomatic and emotional distress (Pearson correlation = 0,46)[29]. Another study with the same population six years later found that SOC was by far the best correlate of health (SCL-90) out of many other variables such as intelligence, energy level, stability, locus of control, mastery and ways of coping[30]. A study involving 162 female critical care nurses found a correlation of –0,296 between SOC and illness, with illness measured with the illness rating scale (SIRS), which included 126 items[31].

In 1993, Antonovsky published a review about correlations with SOC [32] and presented data on the correlation between SOC and measures in the four domains: a global orientation to oneself and environment; stressors; health, illness and well being; and attitudes and behaviour. Several correlations were found between health, illness and well being. The correlations were ranging from non-significant (Cholesterol, triglycerides and glucose, hospital or emergency room contact) to 0,76 (quality of life) and 0,71 (mental health)[32].

## Stress

SOC seems to be highly associated with different measures of stress. A study of 418 adolescents attending a regional high school in Israel found SOC to be negatively related to anxiety response in normal potentially stressful situations, but no relationship was found to acute communal stress[33]. Furthermore, a study that included female critical care nurses showed significant correlations between SOC and event stress (-0,273) and global stress (-0,562)[32]. A study of 302 assistant professors showed that the correlations between SOC and life stress, work stress and general well-being was very strong (0,40; 0,24; 0,62 respectively)[34]. A study that tested the relationship between SOC in early pregnancy and symptom severity of post-traumatic stress disorder (PTSD) and depression after pregnancy loss found a stronger SOC in early pregnancy rendered women somewhat resilient to symptoms of PTSD and depression after pregnancy loss. This appears to be due to the mobilization of crisis support so that crisis support statistically mediated between SOC and PTSD[35]. Another prospective study (N = 95) found that SOC was negatively correlated with life stress, anxiety and depression[36], just as a different prospective study (N = 307) showed a significant correlation between SOC and anxiety when controlling for Global Inventory of Stress (GIS)[33]. Antonovsky showed in a study of two adolescent samples that high negative correlations existed between SOC and trait anxiety (-0,56 and –0,62)[37]. When data from the 1994 Canadian National Population Survey were analysed to investigate the effects of stress, recent traumatic events and social support on SOC, it was shown that all the correlations were significant (chronic stress = -0,53; resent life events = -0,33; perceived social support = 0,16)[38]. Vulnerability to post-traumatic stress disorder in offspring of holocaust survivors has shown to be associated with SOC[39] and so has perceived stress in a population study [23].

### Behaviour

A study of South African patients with essential hypertension found positive associations between self-management and SOC[40]. A Finnish study found that good organizational climate on the job and low job insecurity were related to strong SOC, which was in turn linked to a high level of occupational well-being[41]. For caregivers of demented patients, sense of coherence was a powerful predictor of role overload, since SOC predicted 29% of the variance in role overload. For caregivers to non-demented patients, situation-specific coping responses acted independently of SOC[42]. A low SOC has furthermore shown to be associated with daily drinking[43], alcohol problems[44], food selection[45], physical activity[46] and with suicidal ideation and attempted suicide[47]. In a study among minority women at risk for HIV infection, it was found that those with a weak coherence had more high-risk behaviours than those with a strong coherence[28] and another study concluded that the SOC scale was a beneficial way of identifying vulnerable women in need of increased support during pregnancy[48]. A study of 10,000 children in the five Nordic countries found that poor SOC was more common in the lower social classes and that poor SOC was only slightly associated with child chronic health conditions in general, but that poor SOC was quite common among parents of children with diabetes, epilepsy and psychiatric/nervous problems [49].

## DISCUSSION

The overall picture shows us, that SOC (sense of coherence) is primarily correlated to psychological measures and health measures that incorporate psychological aspects. When it comes to the physical health, only few and merely modest correlations were found. Around half of the studies that investigated the association between SOC and the physical health found an insignificant correlation and in addition we must suspect a certain amount of publication bias. We are aware that all our findings are vulnerable to bias due to systematic failures of other investigators to publish or report certain data (e.g. non-significant results), and that the restriction of our paper to include only published articles may aggravate such bias.

The SOC scale has been used in at least 33 languages in 32 countries[5], which testifies that the instrument is a cross culturally applicable instrument. Besides, from the above mentioned associations, the concept of SOC has among other things been evaluated according to structural sources[50], its relation to meaning, locus of control, learned helplessness and hardiness[51], aging [52] and its relation to modern physics[53]. The concept of SOC has been used in the promotion of different issues such as “how to use the potential of physician-patient-communication to promote health”[54] and “construction of the patients personality profile”[55].

The focus in this paper was to enlighten the association between SOC and different aspects of health. We found that the association to physical health seemed smudged and not as clear as what was stated by Antonovsky. On the other hand, we still believe in the idea of Antonovsky and therefore wish to investigate the route from idea to operationalization in order to find out whether this is adequate. In a future paper we intent to look deeper into the idea, the conceptualization, theory and operationalization behind the sense of coherence scale.

## CONCLUSIONS

Based on this review, divided into the categories: physical health; biological measures; psychological measures; health measures incorporating psychological aspects; stress; and behavioural aspects, we conclude that the sense of coherence (SOC) scale constructed by Aaron Antonovsky is highly correlated with psychological aspects. However, the use of the scale has been unable to show a strong association between SOC and physical health as Antonovsky predicted. We conclude that the sense of coherence, as measured by Antonovsky's SOC scale, is only a weak predictor of physical health, but a very powerful predictor of psychological health, including stress and behavioural aspects. What determines the physical health is thus still not clear.

## ACKNOWLEDGEMENTS

This study was supported by grants from IMK Almene Fond. The quality of life research was approved by the Copenhagen Scientific Ethical Committee under number (KF)V.100.2123/91.

## BIOSKETCHES

**Trine Flensborg-Madsen**, **Bsc,** is a research assistant at the Quality of Life Research Center in Copenhagen, Denmark. She is at this time finishing her master degree in Public Health at the Department of Public Health, University of Copenhagen. E-mail: tfm@livskvalitet.org

**Søren Ventegodt, MD,** is the director of the Nordic School of Holistic Health and Quality of Life Research Center in Copenhagen, Denmark. He is also responsible for a Clinical Research Clinic for Holistic Medicine in Copenhagen and is a popular speaker throughout Scandinavia. He has published numerous scientific or popular articles and a number of books on holistic medicine, quality of life and quality of working life. His most important scientific contributions are the comprehensive SEQOL questionnaire, the very short QOL5 questionnaire, the integrated QOL theory, the holistic process theory, the life mission theory, and the ongoing Danish Quality of Life Research Survey, 1991-94 in cooperation with the University Hospital of Copenhagen and the late professor of pediatrics, Bengt Zachau-Christiansen, MD, PhD. E-Mail: ventegodt@livskvalitet.org. Website: www.livskvalitet.org/

**Joav Merrick, MD, DMSc,** is professor of child health and human development affiliated with the Center for Multidisciplinary Research in Aging, Zusman Child Development Center, Division of Pediatrics and Community Health at the Ben Gurion University, Beer-Sheva, Israel, the medical director of the Division for Mental Retardation, Ministry of Social Affairs, Jerusalem, the founder and director of the National Institute of Child Health and Human Development. Numerous publications in the field of child health and human development, rehabilitation, intellectual disability, disability, health, welfare, abuse, advocacy, quality of life and prevention. Received the Peter Sabroe Child Award for outstanding work on behalf of Danish Children in 1985 and the International LEGO-Prize ("The Children's Nobel Prize") for an extraordinary contribution towards improvement in child welfare and well-being in 1987. E-Mail: jmerrick@internet-zahav.net. Website: www.nichd-israel.com
